# To Our Patrons

**Published:** 1868-02

**Authors:** 


### TO OUR PATRONS.

We send forth with this issue to the patrons of the Journal the January and February numbers in one. We will in future, instead of a monthly, issue a by-monthly. We will save by it, whilst our patrons will loose nothing, as we will give them nearly the same amount of reading matter, original, selected and editorial, at a cost of one-fourth less to them.

All must admit that it is an uphill business to conduct a Medical Journal any where in the Southern States at this time. We are poor indeed; like the balance of our profession, we have suffered by the war, to the extent of all we had. We have had to build houses to live in, and at the same time support our families, and conduct the Journal out of our earnings. How we have done it, we can scarcely tell. "There is a power that tempers the wind to the shorne lamb." It has been so with us. We have gone ahead, and by the grace of God we will continue to do so. It is certainly not in his providence to overthrow Religion, Science and the Arts, twin sisters of progress and civilization, with the temporary decadence of the public liberty. We ask our friends to aid us in bearing our burden, and in the vindication of that Providence which has been our humble reliance in the terrible emergency that has laid our liberties low; overturned the prosperity of a noble people and brought ruin and despotism instead.

To such of our patrons as have paid us, we offer our hearty thanks. To such as have been unable to do so, we tender our sympathies, and beg to assure them that they shall still have the Journal sent to them. To such as have turned their backs upon us, we can only say, *cui malo*, and in the words of the Poet Lauanato, of England, "He that wrongs a friend wrongs himself more, and ever bears in his own bosom a Court of Justice; himself the Judge, and Jury, and prisoner at the bar, ever condemned."

This double number complets volume 8. The March and April number will be the beginning of a new volume, and if we live the Journal will live also, to the end of another year, and another volume.

We ask our contributors to remember us—such of our subscribers as are able to do so, to pay us—and such as are willing to pay when able, to continue their subscriptions and send in their orders. Let us work together to promote the common good, and loosing nothing of our prestige in the glorious past, press on a still more glorious future. We are in the twilight of a new era, let us awake to duty and interest, and prepare to garner the harvest now ready for the sicle.

